# The Wilms' Tumor Suppressor Protein WT1 Is Processed by the Serine Protease HtrA2/Omi

**DOI:** 10.1016/j.molcel.2009.12.023

**Published:** 2010-01-29

**Authors:** Jörg Hartkamp, Brian Carpenter, Stefan G.E. Roberts

**Affiliations:** 1 Faculty of Life Sciences, The Michael Smith Building, University of Manchester, Oxford Road, Manchester M13 9PT, UK; 2 Department of Biological Sciences, University at Buffalo, Buffalo, NY 14260, USA

**Keywords:** PROTEINS, HUMDISEASE

## Abstract

The Wilms' tumor suppressor protein WT1 functions as a transcriptional regulator of genes controlling growth, apoptosis, and differentiation. It has become clear that WT1 can act as an oncogene in many tumors, primarily through the inhibition of apoptosis. Here, we identify the serine protease HtrA2 as a WT1 binding partner and find that it cleaves WT1 at multiple sites following the treatment of cells with cytotoxic drugs. Ablation of HtrA2 activity either by chemical inhibitor or by siRNA prevents the proteolysis of WT1 under apoptotic conditions. Moreover, the apoptosis-dependent cleavage of WT1 is defective in HtrA2 knockout cells. Proteolysis of WT1 by HtrA2 causes the removal of WT1 from its binding sites at gene promoters, leading to alterations in gene regulation that enhance apoptosis. Our findings provide insights into the function of HtrA2 in the regulation of apoptosis and the oncogenic activities of WT1.

## Highlights

► HtrA2 binds to the suppression domain of WT1 and cleaves WT1 at multiple sites ► Treatment of cells with cytotoxic drugs induces HtrA2-dependent cleavage of WT1 ► The proteolysis of WT1 by HtrA2 leads to changes in WT1 target gene expression ► The destruction of WT1 by HtrA2 promotes apoptosis

## Introduction

The Wilms' tumor suppressor protein WT1 plays a central role in the development of several organs and is mutated or aberrantly expressed in pediatric nephroblastoma (reviewed in Rivera and Haber, 2005; Hohenstein and Hastie, 2006). WT1 is expressed as four major isoforms that arise from two alternative splice sites. One inserts 3 amino acids (KTS) between zinc fingers 3 and 4 of WT1. This enhances affinity for RNA and is likely to play a posttranscriptional role. The second alternative splice includes a further 17 amino acids (exon 5) within the transactivation domain. WT1 target genes include growth factors, differentiation markers, cell-cycle regulators, and apoptosis regulators (Rae et al., 2004; Renshaw et al., 2004; Kim et al., 2007, 2009).

The transcriptional regulatory properties of WT1 are complex, and it elicits both activator and repressor functions (reviewed in Roberts, 2005). How WT1 manifests these distinct activities is not clear, but several binding partners of WT1 have been proposed to regulate its function. The ability of WT1 to act dichotomously is achieved through the structure of its N terminus, which is comprised of both a transcriptional activation domain and an adjacent suppression domain (SD) (McKay et al., 1999). The suppression domain acts by recruiting a transcriptional cosuppressor, BASP1 (Carpenter et al., 2004).

Although WT1 functions as a tumor suppressor in the formation of Wilms' tumors, recent findings have shown that wild-type WT1 is expressed in a variety of tumors from different origins that normally do not express WT1 (reviewed in Yang et al., 2007; Oka et al., 2008). Several reports have revealed an antiapoptotic function for WT1, suggesting that WT1 acts as an oncogene in some tumors (Mayo et al., 1999; Richard et al., 2001; Ito et al., 2006; Tatsumi et al., 2008). How WT1 elicits its oncogenic effects is not clear, but several genes that are central to the control of apoptosis have been proposed as targets of WT1, including *Bcl2*, *Bcl2A1*, *Bak*, c-*myc*, and *JunB* (Mayo et al., 1999; Udtha et al., 2003; Han et al., 2004; Renshaw et al., 2004; Morrison et al., 2005; Simpson et al., 2006; Kim et al., 2007; Green et al., 2009).

The serine protease HtrA2/Omi is involved in the regulation of apoptosis and is principally found in the mitochondria, although a fraction is also located in the nucleus (reviewed in Vande Walle et al., 2008). Mice lacking HtrA2 display low body weight, a significant reduction in the size of several organs, and progressive loss of neurons within the striatum of the basal ganglia (Jones et al., 2003; Martins et al., 2004). The proteolytic activity of HtrA2 can be stimulated by several apoptotic stimuli, and substrates include members of the inhibitors of apoptosis family (IAPs) (reviewed in Vande Walle et al., 2008). In this study, we identify the serine protease HtrA2 as a WT1-interacting protein and demonstrate that it can degrade WT1. We show that endogenous WT1 in tumor cells is cleaved following cytotoxic drug treatment and demonstrate that this cleavage is HtrA2 dependent. Our findings suggest that HtrA2 is a critical regulator of WT1 under proapoptotic conditions.

## Results

### HtrA2 Cleaves WT1 In Vitro

We previously mapped the minimal suppression domain of WT1 to a 30 amino acid region juxtaposed to its transcriptional activation domain (McKay et al., 1999; Carpenter et al., 2004). A yeast two-hybrid screen using a HeLa cell cDNA library performed with the suppression domain as bait harvested 59 positive clones, 58 of which encoded the serine protease HtrA2/Omi. A simultaneous screen of the same library with a different region of WT1 (residues 245–297) did not capture HtrA2.

**Figure 1:**
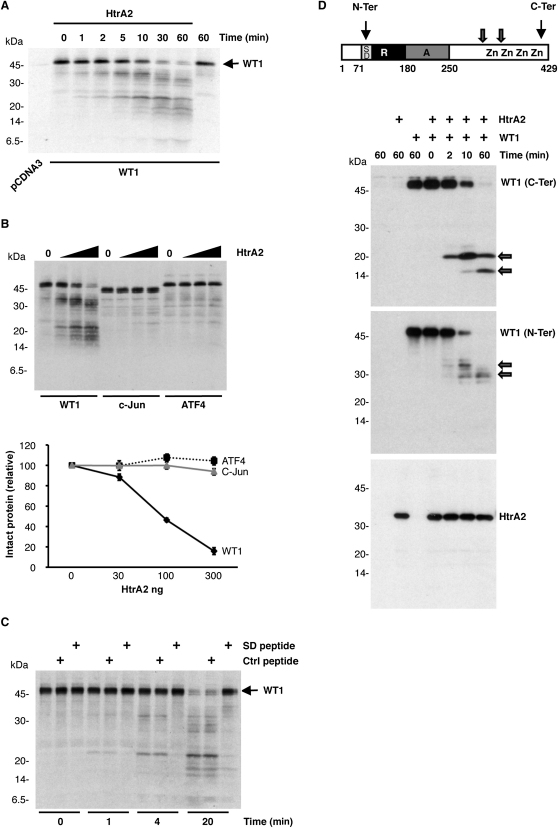
WT1 Is Processed by HtrA2 In Vitro (A) In vitro translated ^35^S-labeled WT1 was incubated for the indicated time with or without recombinant HtrA2 (100 ng). Samples were resolved by SDS-PAGE followed by autoradiography. (B) ^35^S-labeled WT1, c-Jun, and ATF4 were incubated for 30 min with increasing amounts of recombinant wild-type HtrA2 (30, 100, and 300 ng) and analyzed as described in (A). The data are presented graphically below the autoradiogram as remaining intact WT1 with increasing HtrA2 (determined by densitometry). Note that the x axis is not linear. Error bars show standard deviation from the mean (SDM). (C) ^35^S-labeled WT1 was incubated for the indicated time with recombinant wild-type HtrA2 (100 ng) and 1 μg of a synthetic peptide containing the WT1 suppression domain (SD, EQCLSAFTLHSFGQFTGT, residues 86–103 of WT1), a control peptide (MAAPLLHTRLPGDAC), or no peptide and was analyzed as described in (A). (D) In vitro translated WT1 was incubated for the indicated time with recombinant wild-type HtrA2 (100 ng). Samples were immunoblotted with WT1 N-Ter, C-Ter, or HtrA2 antibodies. At top, a diagram of WT1 is shown, indicating the transcriptional regulatory and zinc finger regions. The WT1 N-Ter and C-Ter epitopes and also the approximate locations of HtrA2 cleavage sites are indicated.

To investigate whether HtrA2 can cleave WT1, we synthesized in vitro ^35^S-labeled WT1 (unless otherwise stated, the WT1 isoform used in this study lacks both exon 5 and KTS). ^35^S-labeled WT1 was incubated with recombinant HtrA2 for increasing time periods, and the products were resolved by SDS-PAGE and visualized by autoradiography. As a control, ^35^S-labeled WT1 was incubated under the same conditions in the absence of HtrA2 for the maximum time period. Figure 1A demonstrates that HtrA2 cleaved WT1 at multiple sites in a time-dependent manner. This effect was not due to a general promiscuity of HtrA2 activity in vitro because ^35^S-labeled WT1 was significantly more sensitive to HtrA2 proteolytic activity than were ^35^S-labeled c-Jun or ATF4 (Figure 1B; the data are presented graphically below the autoradiogram). HtrA2 also failed to cleave the WT1 cofactors Par-4 and BASP1 (data not shown).

The yeast two-hybrid screen suggested that the WT1 suppression domain forms a binding site for HtrA2. We therefore tested whether a synthetic peptide containing the minimal WT1 suppression domain (SD) could act as an inhibitor of HtrA2-dependent proteolysis of WT1 in vitro. The SD peptide or a control peptide was incubated with ^35^S-labeled WT1 together with HtrA2. Figure 1C shows that the SD peptide, but not the control peptide, was able to block HtrA2-mediated cleavage of WT1, independently confirming the importance of this domain as an HtrA2-binding site.

Our data so far suggest that HtrA2 can cleave WT1 at multiple sites. To identify the cleavage sites, we next exploited the recognition specificity of two different anti-WT1 antibodies. The F6H2 and C-19 antibodies both bind to single epitopes within WT1. The C-19 antibody recognizes a region very close to the C terminus of WT1 (designated C-Ter), whereas the F6H2 antibody recognizes an epitope within WT1 residues 71–101 (designated N-Ter) (see Figure S1 available online; summarized in the diagram in Figure 1D). WT1, produced by in vitro translation, was subject to HtrA2 treatment as in Figure 1A, but the products were then immunoblotted with either the N-Ter or the C-Ter anti-WT1 antibodies. When immunoblotting with the C-Ter antibody, WT1 degradation by HtrA2 revealed two major proteolytic products of 20 kDa and 15 kDa. As the C-Ter antibody binds at the immediate C terminus of WT1, we can estimate the location of two HtrA2 cleavage sites on WT1 (Figure 1D, top). The same samples immunoblotted with N-Ter antibody revealed major fragment sizes of 35 kDa and 30 kDa, but the cleavage sites cannot be determined specifically because the N-Ter antibody recognizes an internal site in WT1. However, we can conclude that HtrA2 cleaves at, at least, two specific sites in WT1. By comparison of the 20 kDa C-terminal fragment with a WT1 deletion mutant derivative (Δ1–243) (Figure S2), we can further refine the HtrA2 cleavage site to the region of WT1 within residues 280–290. HtrA2 shows a bias to cleave immediately C terminal to nonpolar aliphatic amino acids, with a further preference for hydrophilic residues N terminal to the cleavage site (especially arginine) and a polar residue followed by an aromatic amino acid C terminal to the cleavage site (Martins et al., 2003). We can, therefore, locate the potential cleavage site in WT1 that produces the 20 kDa fragment as Valine-286, which forms a motif (Arg-Arg-Val-Ser-Gly) with 4/5 residues conformity to the HtrA2 sequence preference. A potential cleavage site producing the 15 kDa fragment is Leucine-320, which nests within a motif (Phe-Lys-Leu-Ser-His) with 3/5 residues conformity to the HtrA2 sequence preference.

### HtrA2 Cleaves WT1 in Cells

**Figure 2:**
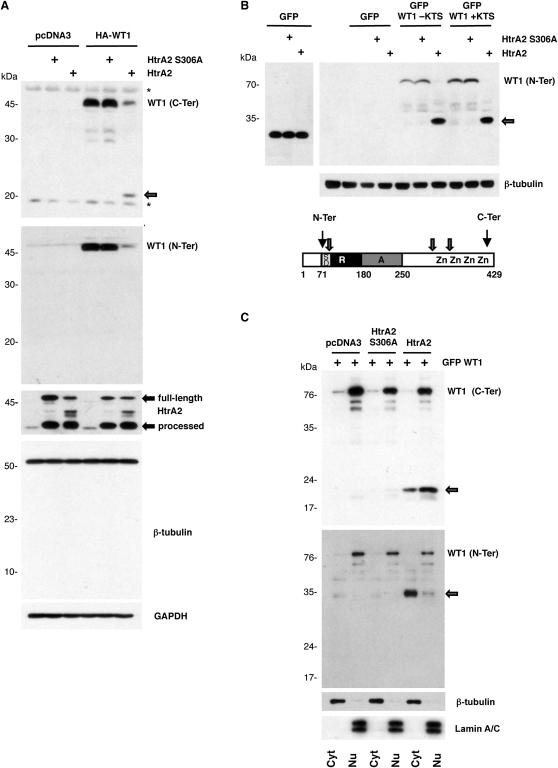
HtrA2 Cleaves WT1 in Cells (A) HeLa cells were cotransfected with plasmids encoding WT1 (1 μg) and vector control, along with plasmid driving expression of either wild-type or the mutant HtrA2 derivative S306A (0.5 μg). At 24 hr after transfection, whole-cell lysates were prepared and immunoblotted with the indicated antibodies. The full-length and processed forms of HtrA2 are indicated. Nonspecific bands are indicated by asterisks. (B) HeLa cells were cotransfected with plasmids encoding GFP-WT1-KTS, GFP-WT1 + KTS, or GFP (1 μg) in combination with vector control, wild-type, or mutant HtrA2 (0.5 μg) and 24 hr after transfection were analyzed as in (A). Below, a diagram of WT1 is shown, indicating the transcriptional regulatory and zinc finger regions. The epitopes recognized by the WT1 N-Ter and C-Ter antibodies and also the approximate locations of HtrA2 cleavage sites are indicated. (C) HeLa cells were transfected as in (B), and then nuclear (Nu) and cytoplasmic (Cyt) extracts were prepared. The samples were immunoblotted with the antibodies indicated. The GFP-WT1 N-terminal and C-terminal proteolytic fragments are indicated.

To investigate whether HtrA2 can cleave WT1 in living cells, we transfected HeLa cells (which do not express endogenous WT1; see Figure S4) with a plasmid driving expression of WT1 along with a plasmid driving expression of either wild-type HtrA2 or an HtrA2 mutant derivative lacking proteolytic activity (HtrA2 S306A). The samples were immunoblotted with the WT1 C-Ter antibodies (Figure 2A, top) or N-Ter antibodies (Figure 2A, bottom), HtrA2 antibodies, and, as controls, β-tubulin and GAPDH antibodies (Figure 2A). Whereas endogenous HtrA2 is primarily detected as the processed active form, overexpression of HtrA2 also results in the production of a full-length form of HtrA2 that remains unprocessed. Coexpression of HtrA2 resulted in cleavage of the overexpressed WT1 and gave rise to a 20 kDa C-terminal cleavage product, whereas the protease-inactive HtrA2 mutant did not. Wild-type HtrA2 and the inactive mutant derivative (S306A) were expressed to equivalent levels (the full-length precursor and processed form are arrowed). We note that the 15 kDa cleavage product that we detected with the assays using in vitro translated WT1 was not observed, suggesting that it is unstable in vivo. Blotting the same samples with N-Ter antibodies confirmed the HtrA2-dependent degradation of WT1, but again, we were unable to observe proteolytic products, suggesting that the N-terminal fragments of WT1 are also unstable in vivo.

To determine the most N-terminal HtrA2 cleavage site in WT1, we used a GFP-WT1 fusion protein in place of native WT1 and immunoblotted with N-Ter antibodies. GFP is not degraded by HtrA2 (Figure 2B, left), but blotting with N-Ter antibodies revealed that GFP-WT1 is proteolyzed by HtrA2, releasing an N-terminal GFP-linked fragment that is ∼9 kDa larger than GFP. Thus, an HtrA2 cleavage site is located close to, or within, the WT1 suppression domain (and also close to the WT1 region that was used as the bait in the yeast two-hybrid screen). A potential cleavage site is Leucine-94, which is within a sequence with 4/5 residue match with the HtrA2 preference. We also tested GFP-WT1 + KTS in the same assay, finding it equally sensitive to HtrA2 cleavage. The forms of WT1 that contain the region encoded by exon 5 are similarly processed by HtrA2 (data not shown). Thus, HtrA2 can cleave all of the major isoforms of WT1 at, at least, three different sites.

WT1 has been reported to shuttle between the nucleus and the cytoplasm (Vajjhala et al., 2003; Niksic et al., 2004). To analyze the location of the HtrA2-dependent WT1 cleavage products, we coexpressed HtrA2 and GFP-tagged WT1 in HeLa cells, prepared cytoplasmic and nuclear extracts, and then immunoblotted with either C-Ter or N-Ter anti-WT1 antibodies (Figure 2C). The rigor of the nuclear/cytoplasmic separation was confirmed by immunoblotting the same samples with anti-β-tubulin (cytoplasmic) and anti-Lamin A/C (nuclear) antibodies. The C-terminal HtrA2 cleavage product of WT1 was distributed between cytosolic and nuclear fraction, whereas most of the WT1 amino-terminal cleavage product localized to the cytosol. These results are consistent with the presence of nuclear localization sequences within the C-terminal zinc finger region of WT1 (see Discenza and Pelletier, 2004).

### HtrA2-Dependent Processing of WT1 Is Induced by Cytotoxic Drugs

**Figure 3:**
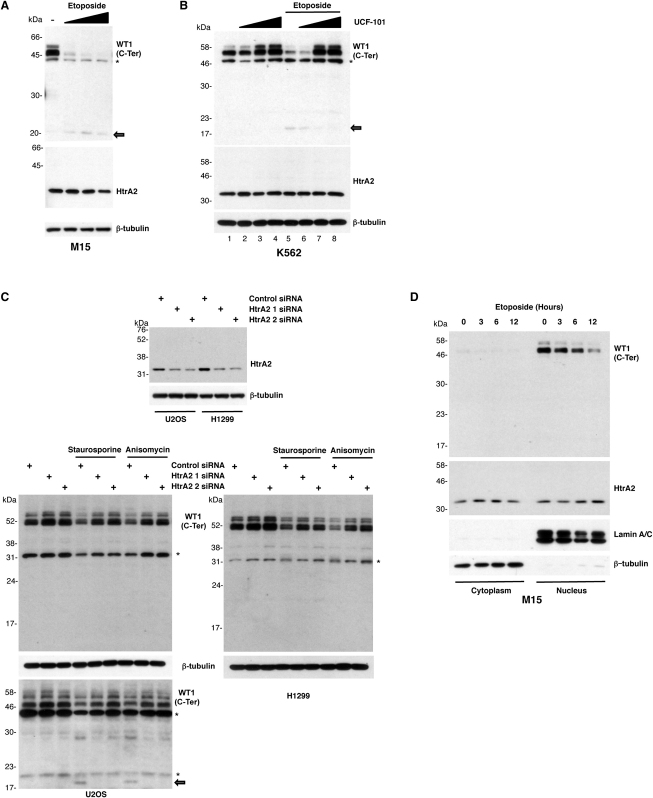
HtrA2 Is Required for the Stimulation of WT1 Cleavage following Treatment with Cytotoxic Drugs (A) Mouse embryonic M15 cells were treated for 20 hr with 10, 30, and 100 μM Etoposide and analyzed by immunoblotting with WT1 antibodies (C-ter). The 20 kDa cleavage product is indicated by an arrow. Nonspecific bands are indicated by asterisks. (B) Human K562 cells were incubated with the HtrA2-specific inhibitor UCF-101 (15, 30, and 45 μM) for 30 min before stimulation with 100 μM Etoposide for 24 hr and were analyzed as in (A). Nonspecific bands are indicated by asterisks. (C) U2OS (left) or H1299 cells (right) were transfected with control siRNA or with two different HtrA2-specific siRNAs. HtrA2 knockdown was confirmed by immunoblotting. At 60 hr after transfection, the cells were stimulated with 0.5 μM Staurosporine or 5 ng/ml Anisomycin for 8 hr and analyzed by immunoblot with the antibodies indicated. The lower immunoblot for the U2OS cells used a different anti-WT1 (C-Ter) antibody that is more sensitive, and the 20 kDa C-terminal proteolytic fragment is indicated. (D) Untreated M15 cells or M15 cells that had been treated with 30 μM etoposide for the time points indicated were fractionated to produce nuclear (Nu) and cytoplasmic (Cyt) fractions. The samples were immunoblotted with the antibodies indicated.

HtrA2 activity in cells can be activated by proapoptotic stimuli, such as the cytotoxic drug Etoposide (reviewed in Vande Walle et al., 2008). We next used the embryonic kidney-derived cell line M15 to test the effect of Etoposide treatment on the integrity of endogenous WT1. Incubation of M15 cells with Etoposide resulted in the processing of full-length WT1 and the accumulation of a 20 kDa C-terminal truncation product (Figure 3A). We observed the same effect when M15 cells were treated with Staurosporine (data not shown). We also note that, with the analysis of endogenous WT1, the signal produced by the 20 kDa C-terminal proteolytic is low compared to the starting level of WT1, suggesting that it is not stable.

As mentioned above, WT1 is expressed in many adult tumors, of which leukemia has received particular attention due to the strong association between WT1 expression posttreatment and poor prognosis (Renshaw et al., 2004; Ariyaratana and Loeb, 2007; Yang et al., 2007). We therefore analyzed the effect of cytotoxic drug treatment on the level of WT1 in the chronic myelogenous leukemic cell line K562. Treatment of K562 cells with Etoposide resulted in the degradation of WT1 and the production of the 20 kDa C-terminal fragment (Figure 3B, compare lanes 1 and 5). To test whether the proteolysis of intact WT1 following cytotoxic drug treatment is HtrA2 dependent, we treated K562 cells with UCF-101, a cell-permeable compound that competitively inhibits HtrA2 protease activity (Cilenti et al., 2003; Klupsch and Downward, 2006). Although UCF-101 has been found to elicit additional cellular responses, HtrA2 is the only enzyme that has been reported as a target of UCF-101 (discussed in Klupsch and Downward, 2006). K562 cells were treated with UCF-101 and 30 min later with Etoposide. Whole-cell extracts were prepared 24 hr later and immunoblotted with C-Ter anti-WT1 antibodies. UCF-101 inhibited the Etoposide-induced degradation of WT1 in K562 cells, suggesting that HtrA2 activity is required for WT1 proteolysis (Figure 3B). Treatment of M15 cells with UCF-101 also prevented the cleavage of WT1 induced by Etoposide (Figure S3).

We next analyzed the effect of cytotoxic drugs on HtrA2-mediated cleavage of WT1 in the tumor-derived cell lines U2OS and H1299, which both express endogenous WT1 (see Figure S4). We transfected H1299 und U20S cells with two different HtrA2 siRNAs, which both elicited efficient ablation of HtrA2 expression (Figure 3C, top), treated the cells with the cytotoxic drugs Staurosporine or Anisomycin, and, 8 hr later, prepared whole-cell extracts, followed by immunoblotting with WT1 C-Ter antibodies. Staurosporine and Anisomycin treatment of H1299 and U2OS cells transfected with control siRNA resulted in significant degradation of endogenous WT1 (Figure 3C). However, the proteolysis of WT1 was completely blocked by the transfection of U2OS or H1299 cells with either of the HtrA2-specific siRNAs. We note that the 20 kDa C-terminal WT1 proteolytic product is only evident when a more sensitive anti-WT1 C-Ter antibody is employed (U20S) (Figure 3C, bottom), further suggesting that the WT1 proteolytic fragment(s) are not stable. However, the production of the 20 kDa WT1 proteolytic product is also specifically prevented by both HtrA2 siRNAs. Thus, ablation of HtrA2 in two different tumor cell lines by siRNA blocks the induction of WT1 cleavage by cytotoxic drugs. We also note that transfection of both the U2OS and H1299 cells with HtrA2 by siRNA raised the resting level of WT1, suggesting that WT1 accumulation is likely to be continually modulated by HtrA2.

As stated above, HtrA2 is present in both the nucleus and cytoplasm. WT1, although principally found in the nucleus, undergoes nucleocytoplasmic shuttling, and a small proportion is therefore present in the cytoplasm. The results of Figure 2C demonstrated that the WT1 fragments generated by HtrA2 are present in both the nucleus and cytoplasm. We were therefore interested to determine the nuclear/cytoplasmic distribution of HtrA2 before and after treatment of cells with a cytotoxic drug. M15 cells were treated with Etoposide for up to 12 hr and subjected to nuclear/cytoplasmic fractionation and immunoblotted with HtrA2, WT1 (C-Ter), Lamin A/C, or β-tubulin antibodies (Figure 3D). HtrA2 was present in both the nucleus and cytoplasm of M15 cells and did not show a significant change in nuclear/cytoplasmic distribution after treatment of the cells with Etoposide (changes in mitochondrial HtrA2 would not be evident in this assay). Consistent with our analysis of GFP-WT1 (Figure 2C), endogenous WT1 in M15 cells was mostly present in the nucleus, with a small fraction observed in the cytoplasm. As before, treatment of the cells with Etoposide resulted in an overall decrease of WT1 but did not alter the relative distribution of WT1 between the nucleus and cytoplasm. Thus, it is likely that WT1 is cleaved by HtrA2 in both the nuclear and cytoplasmic compartments of the cell.

### Upregulation of WT1 in HtrA2 Knockout Mouse Embryonic Fibroblasts

**Figure 4:**
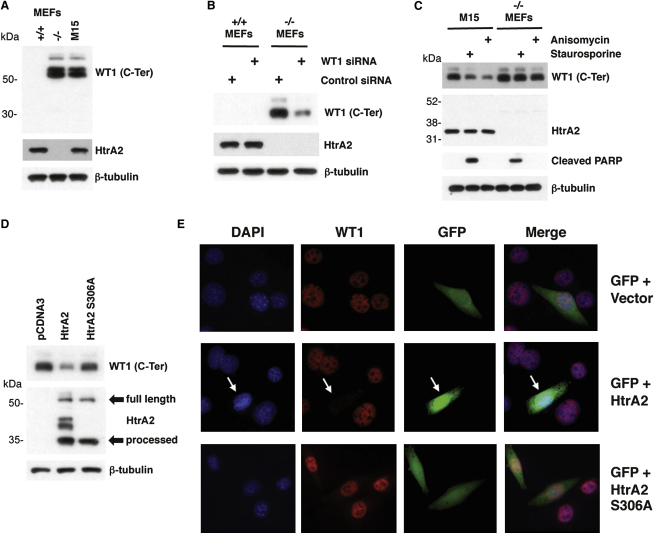
Analysis of WT1 in HtrA2 Null MEFs (A) Whole-cell lysates were prepared from HtrA2^+/+^ or *HtrA2^−/−^* mouse embryonic fibroblasts (MEFs) and compared with mouse M15 cells by immunoblotting. (B) HtrA2^+/+^ or *HtrA2^−/−^* MEFs were transfected with control siRNA or with WT1-specific siRNA, and 30 hr later, whole-cell lysates were prepared and immunoblotted with the antibodies indicated. (C) M15 cells or *HtrA2^−/−^* MEFs were stimulated for 8 hr with 0.5 μM Staurosporine or 5 ng/ml Anisomycin, and whole-cell lysates were prepared and immunoblotted with the antibodies indicated. (D) *HtrA2^−/−^* MEFS were transfected with control vector (pCDNA3), vector driving expression of wild-type HtrA2, or the HtrA2 mutant derivative S306A. Whole-cell lysates were immunoblotted with the antibodies indicated. The full-length and processed forms of HtrA2 are indicated. (E) *HtrA2^−/−^* MEFS were cotransfected with a vector driving expression of GFP along with either control vector (pCDNA3) and vector driving expression of wild-type HtrA2 or the HtrA2 mutant derivative S306A. The cells were analyzed by immunofluorescence with anti-WT1 antibodies (red) and overlayed with the GFP signal.

So far, we have determined that ablation of HtrA2 activity either by a chemical inhibitor or by transfection of HtrA2 siRNA prevents the degradation of endogenous WT1 following the treatment of cells with cytotoxic drugs. We next obtained the previously described HtrA2 knockout (*HtrA2^−/−^*) mouse embryonic fibroblasts (MEFs) and corresponding wild-type (*HtrA2^+/+^*) MEFs (Martins et al., 2004). Whole-cell extracts were prepared from *HtrA2^+/+^* MEFs, *HtrA2^−/−^* MEFs, and mouse M15 cells; resolved by SDS-PAGE; and immunoblotted with anti-WT1 antibodies (C-Ter). Surprisingly, the *HtrA2^−/−^* MEFs expressed WT1 to a level similar to that observed of M15 cells, whereas WT1 was not detected in the *HtrA2^+/+^* MEFs (Figure 4A). The expression of WT1 in the *HtrA2^−/−^* MEFs was confirmed by treatment with WT1 siRNA, followed by immunoblotting with C-Ter anti-WT1 antibodies (Figure 4B). Analysis of WT1 mRNA revealed that the expression of WT1 was at least partly attributable to the upregulation of WT1 message in the *HtrA2^−/−^* MEFs (Figure S5).

As the *HtrA2^−/−^* MEFs express WT1, we next determined the effect of Staurosporine or Anisomycin treatment on the level of endogenous WT1 protein compared to the treatment of M15 cells with the same agents. Figure 4C shows that both Staurosporine and Anisomycin treatment lead to the degradation of WT1 in M15 cells. In contrast, the effect of both Staurosporine and Anisomycin on WT1 protein level was significantly reduced in the *HtrA2^−/−^* MEFs. Importantly, this was not due to an impaired response of the *HtrA2^−/−^* MEFs to the cytotoxic agents, as the treatment of both *HtrA2^+/+^* and *HtrA2^−/−^* MEFs with Staurosporine resulted in PARP cleavage, which serves as a marker for cells undergoing apoptosis. Anisomycin treatment of both cells also elicited PARP cleavage, but this occurred at a later time point and is thus not evident in the figure (data not shown).

To determine whether the reintroduction of HtrA2 into the *HtrA2^−/−^* MEFs would lead to degradation of WT1, *HtrA2^−/−^* MEFs were transfected with a plasmid driving expression of either wild-type HtrA2 or the protease-defective HtrA2 mutant derivative (S306A). Whole-cell lysates were prepared and immunoblotted with C-Ter WT1 antibodies, HtrA2 antibodies, or β-tubulin antibodies (Figure 4D). The introduction of wild-type HtrA2, but not the inactive mutant derivative (S306A), into the *HtrA2^−/−^* MEFs resulted in cleavage of the endogenous WT1. In addition, we transfected the *HtrA2^−/−^* MEFs with GFP along with either control expression vector or the same vector driving HtrA2 expression and performed immunofluorescence to detect both the endogenous WT1 and the transfected GFP (Figure 4E). Transfection of HtrA2, but not the HtrA2 S306A mutant derivative, resulted in a significant reduction of endogenous WT1, as demonstrated by the cofluorescence with GFP. Taken together, these results demonstrate that loss of HtrA2 leads to the accumulation of WT1 that can only be reversed by the reintroduction of catalytically active HtrA2.

### WT1 Expression Confers Resistance to Apoptosis Induced by Cytotoxic Drugs

**Figure 5:**
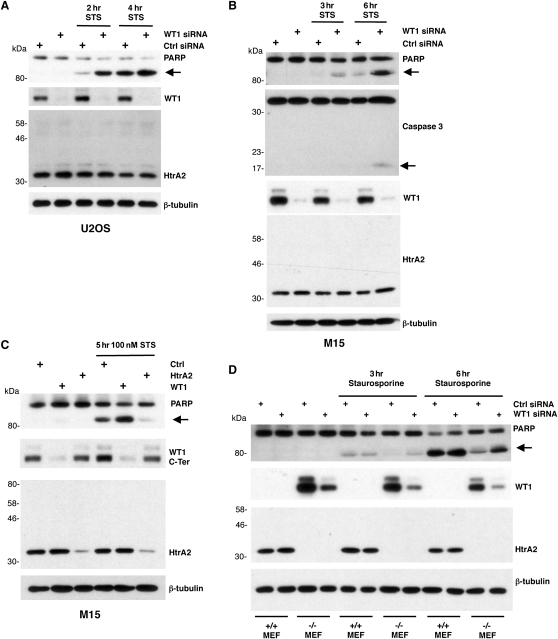
WT1 Is an Inhibitor of Apoptosis (A) U2OS cells were transfected with a control siRNA or siRNA that targets WT1. At 24 hr later, the cells were treated with 100 nM Staurosporine for the indicated times. Whole-cell extracts were prepared and immunoblotted with the antibodies indicated. (B) M15 cells were analyzed as in (A) except that the siRNAs were mouse specific. The samples were also immunoblotted with caspase 3 antibody. (C) M15 cells were transfected with control or HtrA2 siRNA, and 24 hr later, were split 1:4. A further 24 hr later, the cells were transfected with control or WT1 siRNA, and then 24 hr later, they were treated with 100 nM Staurosporine for 5 hr and harvested, and whole-cell extracts were subjected to immunoblotting with the antibodies indicated. (D) MEFs were transfected with control or WT1-specific siRNA, and 24 hr later, they were treated with 100 nM Staurosporine for the indicated time, and then whole-cell lysates were prepared and analyzed by immunoblotting with the antibodies indicated.

Several recent studies have suggested that WT1 can inhibit apoptosis (Mayo et al., 1999; Richard et al., 2001; Ito et al., 2006; Tatsumi et al., 2008). We therefore analyzed the effect of WT1 ablation by siRNA on the ability of cytotoxic drugs to induce apoptosis. U2OS cells were transfected with control siRNA or WT1 siRNA, and then the cells were treated with Staurosporine and whole-cell extracts were prepared. Importantly, the time points were selected to analyze the cells before WT1 proteolysis was evident to ensure that the ablation of WT1 was only due to the siRNA. The whole-cell extracts were subject to immunoblotting to detect WT1, HtrA2, β-tubulin, and, as a marker of apoptosis, PARP (Figure 5A). Ablation of WT1 expression by siRNA significantly enhanced PARP cleavage induced by Staurosporine, suggesting that WT1 acts in an antiapoptotic fashion in U2OS cells. We observed the same antiapoptotic effect of WT1 in M15 cells, in which we analyzed both PARP and caspase 3 cleavage as markers of apoptosis (Figure 5B).

We next compared the roles of WT1 and HtrA2 in the regulation of apoptosis in M15 cells following exposure to Staurosporine (Figure 5C). As above, ablation of WT1 by siRNA enhanced the cleavage of PARP induced by Staurosporine. HtrA2 ablation by siRNA elicited the opposite effect and reduced the cleavage of PARP, consistent with several previous studies (reviewed in Vande Walle et al., 2008). Thus, WT1 and HtrA2 act in an antiapoptotic and proapoptotic fashion, respectively. We next analyzed the *HtrA2^+/+^* and *HtrA2^−/−^* MEFs to determine the effect of WT1 siRNA on PARP cleavage. The *HtrA2^−/−^* MEFs, which express WT1, showed a significantly lower level of PARP cleavage at both 3 and 6 hr following Staurosporine exposure. These results are consistent with the proapoptotic function of HtrA2. Significantly, transfection of the *HtrA2^−/−^* MEFs, but not the wild-type MEFs, with WT1 siRNA resulted in an increase in apoptosis induced by Staurosporine. Taken together, the data of Figure 5 suggest that WT1 acts to inhibit apoptosis but that this can be negated by the cleavage of WT1 by HtrA2.

### A Role for HtrA2-Dependent Processing of WT1 in Transcriptional Regulation under Apoptotic Conditions

**Figure 6:**
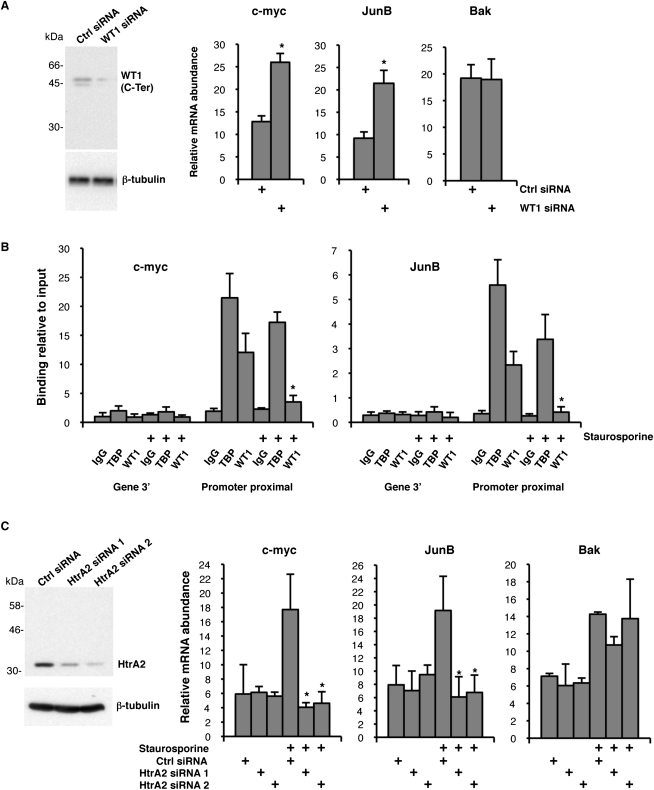
Degradation of WT1 by HtrA2 Releases the c-*myc* and *JunB* Genes from Transcriptional Repression by WT1 (A) U2OS cells were transfected as in Figure 5A and either used to prepare whole-cell extracts for immunoblotting with the indicated antibodies or to prepare total RNA and then cDNA. The expression of c-*myc*, *JunB*, and *Bak* are shown graphically relative to GAPDH expression. Error bars show standard deviation from the mean (SDM). ^∗^p < 0.05 in Student's t test. (B) U2OS cells were subject to ChIP with the antibodies indicated before and after treatment with 0.5 μM Staurosprine for 12 hr. Binding of WT1 and TBP to the c-*myc* (left graph) and *JunB* (right graph) promoters or the 3′ gene regions was quantitated by PCR and expressed as percent enrichment of the input chromatin. Error bars show SDM. ^∗^p < 0.05 in Student's t test. (C) U2OS cells were treated with 200 nM Staurosporine following transfection with either control siRNA or two different HtrA2-specific siRNAs. Total RNA was prepared and quantitative PCR was performed to analyze c-*myc*, *JunB*, and *Bak* expression relative to GAPDH. Error bars show SDM. ^∗^p < 0.05 in Student's t test. An immunoblot of HtrA2 is shown at left, confirming robust knockdown of HtrA2 by the siRNAs.

Our data so far suggest that WT1 is a bona fide substrate of HtrA2 that is cleaved under proapoptotic conditions. We next explored the consequences of HtrA2-dependent WT1 proteolysis on the expression of WT1 target genes. WT1 exhibits cell context-dependent transcriptional regulatory activity due to modulation by cofactors (reviewed in Roberts, 2005). We therefore analyzed the expression of various previously proposed WT1 target genes that also play a role in apoptosis. U2OS cells were transfected with a control siRNA or a siRNA targeting WT1, and 24 hr later, RNA was prepared and cDNA produced. Blotting of simultaneously prepared whole-cell extracts with anti-WT1 antibodies confirmed robust depletion of WT1 by siRNA (Figure 6A, left). Quantitative PCR was used to analyze the effect of WT1 depletion on a selection of WT1 target genes that have been proposed to play a role in apoptosis. The results obtained for c-*myc*, *JunB*, and *Bak* are shown in Figure 6A. c-*myc* and *JunB* mRNA were elevated when WT1 was depleted in U2OS cells, whereas *Bak* mRNA was not affected. Thus, in U2OS cells, c-*myc* and *JunB* are subject to transcriptional repression by WT1. However, unlike in Saos2 cells (Morrison et al., 2005) and murine podocyte cells (Green et al., 2009), *Bak* does not appear to be regulated by WT1 in U2OS cells, at least under the conditions tested here.

We next used chromatin immunoprecipitation (ChIP) to determine whether WT1 is bound to the c-*myc* and *JunB* promoters in U2OS cells and also whether binding is perturbed following HtrA2 activation. Control U2OS cells or cells treated with Staurosporine were subjected to ChIP with anti-WT1 (C-Ter) antibodies, anti-TBP (TATA-binding protein) antibodies (as a positive control), or rabbit IgG (as a negative control) (Figure 6B). The c-*myc* and *JunB* promoter regions (which contain both the WT1-binding sites and core promoter regions; see Experimental Procedures) were amplified and compared with amplification of a control region from the 3′ end of each gene. In resting U2OS cells, WT1 and TBP were located at the promoter region of both the c-*myc* and *JunB* genes, but not at the 3′ regions of either gene. Treatment of U2OS cells with Staurosporine resulted in a significant decrease in the occupancy of both the c-*myc* and *JunB* promoters by WT1, but not by TBP. Thus, following treatment of U2OS cells with Staurosporine under conditions in which WT1 is degraded by HtrA2 results in the loss of WT1 binding to the c-*myc* and *JunB* promoters.

Our data so far suggest that WT1 actively represses transcription of the c-*myc* and *JunB* genes, but not the *Bak* gene, in resting U2OS cells. However, following exposure to Staurosporine, WT1 is released from the c-*myc* and *JunB* promoters. We next determined the effect of Staurosporine treatment of U2OS cells on the expression of c-*myc*, *JunB*, and *Bak*. Staurosporine treatment of U2OS cells resulted in a significant increase in c-*myc* and *JunB* mRNA (Figure 6C), suggesting that the proteolysis of WT1 results in derepression of the c-*myc* and *JunB* genes. Significantly, this effect was completely blocked when the cells had first been transfected with either of two HtrA2-specific siRNAs, but not a control siRNA. As observed with c-*myc* and *JunB*, *Bak* expression was also elevated following treatment with Staurosporine. However, in agreement with our finding that WT1 does not appear to regulate *Bak* in U2OS cells, the induction of *Bak* was not significantly affected by either HtrA2 siRNA. Taken together, the data in Figure 6 suggest that repression of the c-*myc* and *JunB* promoters by WT1 in U2OS cells is relieved by the HtrA2-mediated degradation of WT1 following cytotoxic drug treatment.

## Discussion

Several previous studies have reported a role for WT1 in the regulation of apoptosis. WT1 can elicit both pro- and antiapoptotic effects, the latter function being consistent with the oncogenic activities of WT1. Thus, when WT1 acts as an oncogene, the activation of HtrA2 by proapoptotic stimuli and subsequent degradation of WT1 might facilitate the clearance of tumorigenic cells. Indeed, tumors that retain high expression of WT1 following chemotherapy generally result in a poor outcome (reviewed in Yang et al., 2007). Moreover, clinical trials using peptide vaccines against WT1 in patients with leukemia, breast, and lung cancer are showing promising therapeutic effects (Oka et al., 2008). Thus, the aberrant expression of WT1 plays a critical role in either or both the transformation process and the maintenance of the transformed phenotype. HtrA2-mediated proteolysis of WT1 might, therefore, directly contribute to the effect of chemotherapeutic drugs in the treatment of WT1-dependent tumors.

Our data suggest that c-*myc* and *JunB* might be important target genes in the regulation of apoptosis by WT1 in U2OS cells. However, further investigation will be required to decipher the complex interplay of the WT1 target genes that are altered in expression following proteolysis of WT1. Moreover, it is not clear whether the C-terminal proteolytic fragment of WT1 (which retains intact zinc fingers) performs any regulatory role. The C-terminal fragment does not appear to be stable, but this does not exclude a transient function or that, under some circumstances, it might be stabilized. Our ChIP experiments were performed with an antibody that recognizes the C-terminal proteolytic fragment, suggesting that it does not significantly associate with the c-*myc* or *JunB* promoters. However, several WT1 interaction partners bind to the zinc finger region (reviewed in Roberts, 2005), and thus a role for the C-terminal fragment cannot yet be excluded. We were also not able to detect HtrA2 association with the c-*myc* or *JunB* promoters (data not shown) but cannot exclude the possibility of a transient association that was not captured by ChIP.

A surprising finding in this study was that the *HtrA2^−/−^* MEFs showed a marked elevation in WT1 mRNA and protein when compared to the wild-type MEFs. This raises the intriguing possibility of a feedback loop in WT1 expression that will require further investigation. Indeed, the WT1 gene has previously been reported to autoregulate (Malik et al., 1994; Rupprecht et al., 1994). It is very unlikely, however, that HtrA2 null mice exhibit a general increase in WT1 expression; any feedback loop would probably require other factors and thus exhibit tissue specificity. Further insights into this possibility will be gained by analysis of WT1 expression in the HtrA2 knockout mice.

A recent large-scale study analyzing more than 1000 different cellular proteins identified only 15 HtrA2 substrates, suggesting that HtrA2 exhibits a high degree of specificity (Vande Walle et al., 2007). β-tubulin was reported as a potential substrate of HtrA2. However, in our analyses, we did not observe a significant reduction of β-tubulin following either overexpression of HtrA2 or the treatment of cells with cytotoxic drugs. In vitro studies that characterized specific HtrA2 binding and cleavage sites using synthetic peptides reported a modest preference in target amino acid sequences (Martins et al., 2003). Considering the high degree of HtrA2 specificity observed in vivo, it is likely that HtrA2 processing of its substrates is tightly regulated by subcellular localization or modulatory/targeting proteins.

Several interaction partners of WT1 have been reported, and it will therefore be of interest to analyze their capacity to regulate the processing of WT1 by HtrA2. WT1 cofactors that block HtrA2-mediated processing of WT1 could potentially provide a switch that regulates the dichotomy of the tumor suppressor and oncogenic properties of WT1. HtrA2-mediated processing of WT1 might also play a role in development. HtrA2 null mice exhibit a general reduced organ size, which includes many sites of WT1 expression and known requirement for WT1 in development (Martins et al., 2004). Further studies will determine how the processing of WT1 is regulated by WT1 cofactors and the role that they play in the modulation of WT1 activity in development and disease.

## Experimental Procedures

### Cells and Antibodies

Human embryo kidney (HEK) 293, HeLa, U2OS, and M15 cells were cultured in DMEM supplemented with 10% fetal bovine serum (FBS). H1299 and K562 cells were cultured in RPMI supplemented with 10% FBS and 2 mM L-Glutamine. MEFs derived from wild-type and *HtrA2^−/−^* mice were cultured as described previously (Martins et al., 2004). HEK293 and HeLa cells were transiently transfected using Lipofectamine 2000 (Invitrogen) according to the manufacturer's guidelines. MEFs were transfected by electroporation using the Amaxa Nucleofector, program A-23, and MEF solution 2 (Amaxa Biosystems).

HeLa, U2OS, and H1299 cells were transfected with siRNA oligos against human HtrA2 (Ambion; Cat#AM16706) or (5′-3′: AACGCTGAGGATTCAGACTAATT) (QIAGEN), human WT1 (Santa Cruz), and Silencer Negative control #1 (Ambion) as control at a final concentration of 30 nM using Oligofectamine (Invitrogen). M15 cells were transfected with siRNA oligos against mouse WT1 (Santa Cruz), mouse HtrA2 (5′-3′: CACACTGAGGATTCAAACCAATT) (QIAGEN), and Silencer Negative control #1 (Ambion) at a final concentration of 30 nM. For HtrA2 analysis, cells were analyzed after 60 hr, whereas for WT1 analysis, cells were analyzed 24 hr after transfection and harvested for western blotting. Delivery of siRNAs into MEFs was performed using the Amaxa nucleofector. Cells were nucleofected with either Silencer Negative control #1 as control or siRNA against mouse WT1 (Santa Cruz) at a final concentration of 20 nM. After 30 hr, cells were harvested for western blotting.

Anti-WT1 (C19), anti-HtrA2 (V-17), anti-GAPDH (6C5), and anti-Lamin A/C (N-18) were from Santa Cruz Biotechnologies; anti-WT1 (F6H2) was from Dako; anti-WT1 (Wilms tumor) was from NeoMarkers; β-tubulin (TAT1) was from Cancer Research UK; and cleaved PARP (Asp214), PARP, and caspase 3 were from Cell Signaling. TBP antibodies were from Abcam, and control IgG was from Millipore. The synthetic SD and control peptides have been described before (Carpenter et al., 2004). MEFs were transfected and plated onto glass coverslips. At 24 hr after transfection, cells were fixed in 4% paraformaldehyde, and for immunostaining, cells were probed with anti-WT1 (F6H2) antibody (1:100) and Alexa Fluor 594-conjugated anti-mouse secondary antibody.

### Plasmids and Proteins

The expression construct pCDNA3 HtrA2 was generated by amplification of the *HtrA2* cDNA and subcloned into pCDNA3. PCDNA3 HtrA2 S306A was generated by site-directed mutagenesis with the QuickChange kit (Stratagene) and confirmed by DNA sequencing. PCDNA3 GFP WT1 – KTS and pCDNA3 GFP WT1 + KTS were a gift from A. Ward (Dutton et al., 2006). Plasmids encoding c-*jun* and ATF4 were a kind gift of Michael Green. Recombinant human HtrA2 was from R&D Systems. Etoposide, Anisomycin, and UCF-101 were from Calbiochem, and Staurosporine was from Sigma.

The HeLa library yeast two-hybrid system was the Matchmaker kit from Clontech. The WT1 suppression domain (residues 71–101) was cloned in frame to the GAL4 DNA-binding domain in the vector. WT1, c-Jun, and ATF4 were in vitro translated using the TNT Quick kit from Promega. WT1 cleavage was assayed by incubating 100 ng of recombinant HtrA2 with in vitro translated substrate at 37°C in cleavage buffer (50 mM HEPES [pH 7.5], 50 mM NaCl, 1 mM EDTA, 5 mM DTT, and 10% glycerol) and terminated by the addition of SDS-PAGE loading dye. Nuclear/cytoplasmic extraction was performed using the Nuclear Extract Kit from Active Motif.

### RNA Analysis and Chromatin Immunoprecipitation

Total RNA was prepared using the QIAGEN RNeasy kit. cDNA was prepared using either the Promega Reverse Transcription kit or BioRad iScript cDNA synthesis kit. Real-time PCR was performed using a BioRad MiniOpticon System and SYBR Green assay reagents. Primers for c-*myc* mRNA analysis were: forward, 5′-GCCACGTCTCCACACATCAG-3′ and reverse, 5′-TGGTGCATTTTCGGTTGTTG-3′. For GAPDH, forward, 5′-ACAGTCAGCCGCATCTTCTT-3′ and reverse, 5′-ACGACCAAATCCGTTGACTC-3′. For *JunB*, forward, 5′-TGGTGGCCTCTCTCTACACGA-3′ and reverse, 5′-GGGTCGGCCAGGTTGAC-3′. For *Bak*, forward, 5′-GAACAGGAGGCTGAAGGGGT-3′ and reverse, 5′-TCAGGCCATGCTGGTAGACG-3′. Annealing temperatures were 60°C for c-*myc*, *Bak*, and *JunB* and 55°C for GAPDH.

ChIP assays were performed as described (Nelson et al., 2006). Primers for the amplification of the c-*myc* promoter were: forward, 5′-TCAAACAGTACTGCTACGGA-3′ and reverse 5′-AGAGCCGCATGAATTAACTA-3′ and for a control region downstream of the c-*myc* gene, forward, 5′-AGAACTGCTAAACCAGAATGT-3′ and reverse, 5′-AGACAGGGTCTCACCATCT-3′. Primers for the amplification of the *JunB* promoter were: forward, 5′-GGTCCTGGTATTTGTCCCAG-3′ and reverse 5′-CTCGCGTCACTGTCAGGAAG-3′ and for a control region downstream of the *JunB* gene, forward, 5′-CAGCTCAGTGCTGTTGGTGG-3′ and reverse, 5′-ACCATCCAACCCTGGAGATC-3′. Annealing temperature was 52°C.

## Supplemental Information

Document S1. Five Figures
