# Supplementary material for: The Wilms' Tumor Suppressor Protein WT1 Is Processed by the Serine Protease HtrA2/Omi
Source: Mol Cell. 2010 Jan 29;37(2):159–71. doi: 10.1016/j.molcel.2009.12.023 (PMC2815029; doi:10.1016/j.molcel.2009.12.023)
Supplement: Document S1. Five Figures [file mmc1.pdf]

Molecular Cell, *Volume 37*

Supplemental Information

The Wilms' Tumor Suppressor Protein WT1 Is Processed by the Serine Protease HtrA2/Omi

Jörg Hartkamp, Brian Carpenter, and Stefan G.E. Roberts

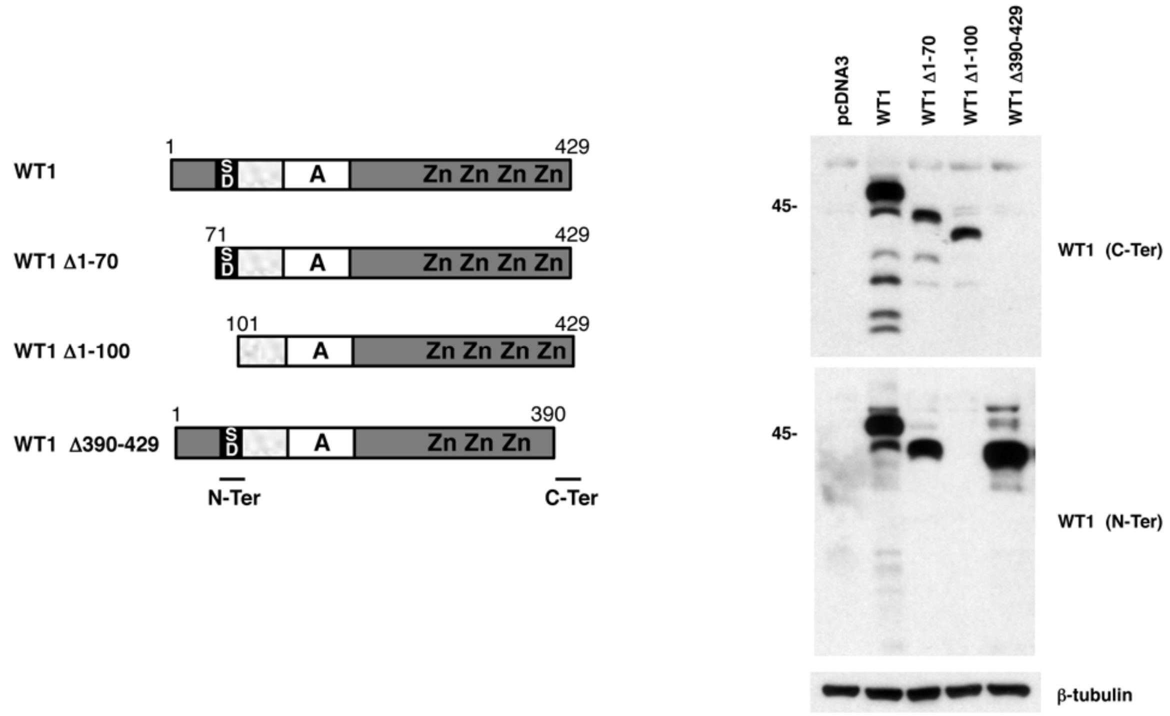

Supplemental Figure 1. Determination of the WT1 epitopes recognised by the N-Ter and C-Ter anti-WT1 antibodies.

Plasmids driving the expression of the indicated WT1 deletion mutant derivatives were transfected into embryonic kidney 293 cells. Whole cell lysates were prepared and subject to immunoblotting with anti-WT1 antibodies (N-Ter and C-Ter) or anti-β-tubulin antibodies. The epitopes recognised by the N-Ter and C-Ter anti-WT1 antibodies are indicated.

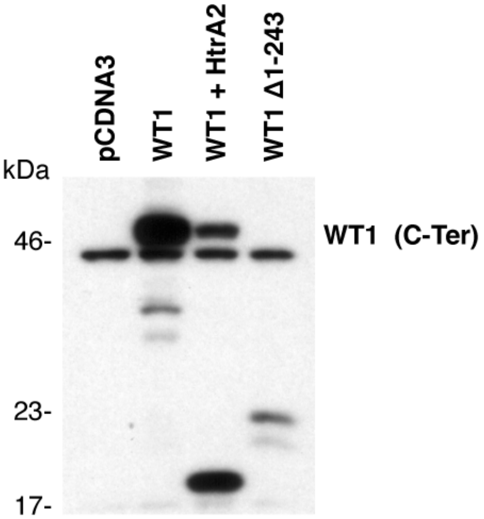

Supplemental Figure 2. Refinement of the location of the major C-terminal WT1 cleavage site.
Plasmids driving the expression of wild type WT1or the deletion mutant derivative Δ1-243 were transfected into HeLa cells. A plasmid driving HtrA2 expression was cotransfected where indicated. Whole cell lysates were prepared and subject to immunoblotting with anti-WT1 antibodies (C-Ter).

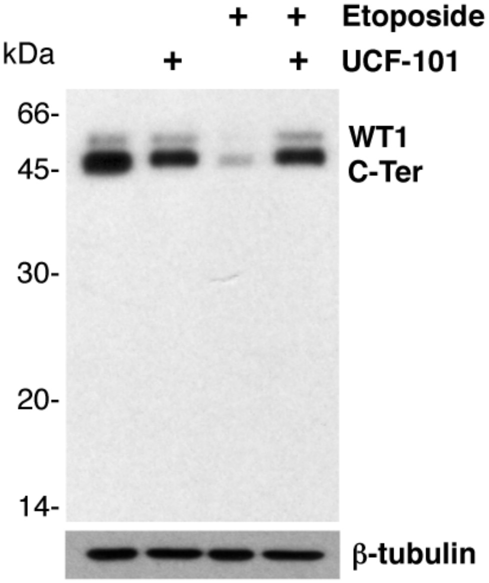

Supplemental Figure 3. UCF-101 inhibits proteolysis of WT1 induced by Etoposide in M15 cells. M15 cells were incubated with the HtrA2 specific inhibitor UCF-101 (50 mM) for 30 minutes before stimulation with 30 mM Etoposide for 12 hours. Whole cell extracts were subject to immunoblotting with anti-WT1 antibodies.

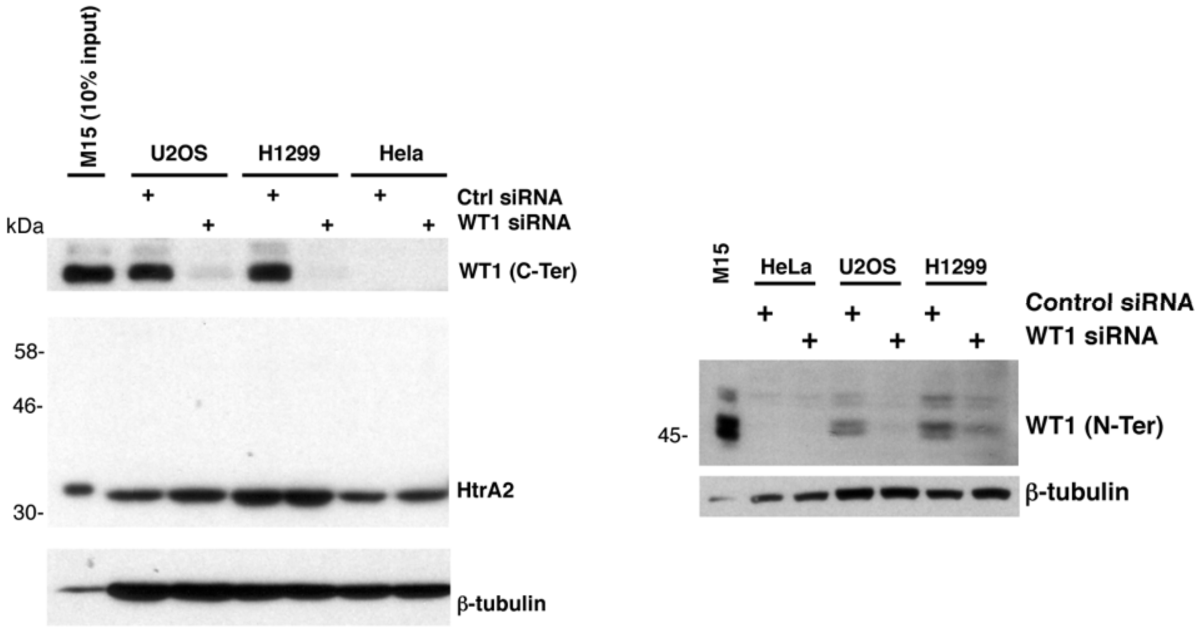

Supplemental Figure 4. U2OS and H1299 cells express endogenous WT1. HeLa, U2OS and H1299 cells were transfected with 20 nM of either a control siRNA or siRNA recognising WT1 mRNA. Whole cell lysates were prepared 24 h after transfection and immunoblotted with anti-HtrA2, WT1 C-Ter antibodies (left), N-Ter antibodies (right) and β-tubulin antibodies. 10% of a M15 lysate was used as a control for WT1 expression.

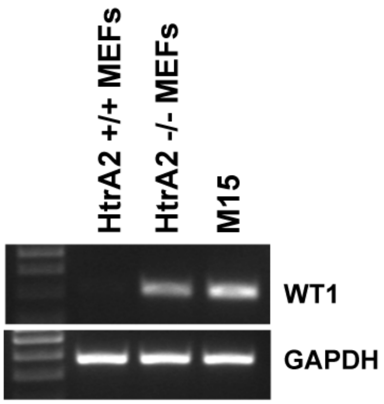

Supplemental Figure 5. HtrA2 -/- MEFs express WT1. Total RNA was prepared from control HtrA2 +/+ MEFs, HtrA2 -/- MEFs and M15 cells. WT1 mRNA was amplified by semiquantitative RT-PCR and resolved by agarose gel electrophoresis.
Primers for WT1: Forward, 5'-GAGACA(CT)ACAGGTGTGAAACCATT-3',
Reverse, 5'-GCCA(GC)(GC)TGGAGTTTGGTCA-3'
Primers for GAPDH: Forward, 5'-ACAGTCAGCCGCATCTTCTT-3', Reverse, 5'-GACAAGCTTCCCGTTCTCAG-3'.
